# 

**Published:** 1856-04

**Authors:** 


ARTICLE I.—Tracheotomy for the removal of a Foreign Body. By M. G. SLAUGHTER, M. D., Pinckneyville, Ala., ... 449

ARTICLE II.—Report of a Case of Placenta Prævia, and one of Shoulder Presentation. By S. W. BURNEY, M. D., of Forsyth, Ga. ... 451

ARTICLE III.—Difficulty and Importance of correct Diagnosis, and especially in certain Nervous Affections. By J. WASHINGTON JONES, M. D., of Auburn, Ala., ... 455

ARTICLE IV.—Operation of Tracheotomy in Croup. By E. J. ROACH, M. D. of Atlanta, Ga., ... 459

ARTICLE V.—Extract from the Records of the Atlanta Medical Society. By EBEN HILLYER, M. D., Secretary. Feb. 28, 1856. ... 460

### *SELECTIONS.*

Remarks on the Diagnosis of Dislocations of the Shoulder Joint, ... 465

Letter from Paris, ... 468

Description of a Case of Frontal Pain occurring in a patient in the Marine Hospital, Charleston, S. C., relieved by large doses of Hydriodate of Potassa, ... 472

Report of a Committee to consider and report on the subject of Home Adulterations, ... 475

Observations on the Rigidity of the Os Uteri and Perinæum, ... 477

On Varioloid and Varicella, ... 479

On the Prevention of Pitting in Smallpox, ... 481

Excision of the Cervix Uteri for Corroding Ulcers, ... 483

An overdose of Homœpathy, ... 489

Attempt at Self-castration, ... 490

Case of Triplets, with an unusual shortening of one of the cords ... 491

### *EDITORIAL AND MISCELLANEOUS.*

Epidemical and Local Influences on disease, ... 493

Bibliographical, ... 496

Chloroform in Labor, ... 499

"The Monthly Stethescope and Medical Reporter." ... 504

Circular.—American Medical Association, ... 505

State Medical Society, ... 506

On the contagiousness of Hooping-cough, ... 506

A Case of Symphyseotomy, ... 507

Tracheotomy in childhood, ... 507

Formulæ for the Internal Administration of Chloroform, ... 508

On the destruction of Non-vascular Nœvi by the Vienna Caustic, ... 509

Pharmacy in Baltimore, ... 510

Cultivation of Liquorice in this country ... 511

The last Phase of Quackery, ... 512

Quick Process for Mercurial Ointment, ... 512

